# Data on Vulnerability Detection in Android

**DOI:** 10.1016/j.dib.2018.12.038

**Published:** 2018-12-15

**Authors:** Shivi Garg, Niyati Baliyan

**Affiliations:** Department of Information Technology, Indira Gandhi Delhi Technical University for Women, Delhi, India

## Abstract

The data in this article have been collaborated from mainly four sources- Google Playstore,^1^ Wandoujia^2^ (third party app store market), AMD^3^ and Androzoo.^4^ These data include ~85,000 APKs (Android Package Kit), both malicious and benign from these data sources. Static and dynamic features are extracted from these APK files, and then supervised machines learning algorithms are employed for malware detection in Android. This data article also provides the Python code for data analysis. For feature extraction, a generic algorithm has also been incorporated, thereby, selecting important and relevant feature subset. Conclusive results obtained from this data set are further comprehended and interpreted in our latest research study “A Novel Parallel Classifier Scheme for Vulnerability Detection in Android” (Garg et al., 2018). This proved to be precious contribution for ensembling classifiers in machine learning to detect malware in Android.

**Specifications table**

**Table:** 

Subject area	Computer Science Applications
More specific subject area	Data Mining and Machine Learning
Type of data	Figures, Tables, Python Code, Malicious and Benign APK feature set
How data were acquired	Data were acquired using Google Play store and Wandoujia app market (APKPure to download the APKs), AMD and Androzoo. Data set can be accessed using the links given below:
	Google playstore: https://play.google.com/store/apps
	Wandoujia app market: http://www.wandoujia.com/apps
	AMD: http://amd.arguslab.org/sharing
	Androzoo: https://androzoo.uni.lu/access
Data format	Data are processed using Python (*.py) format
Experimental factors	Configuration for Android Emulator
	Platform used- Android Studio 1.5.1
	Device- Nexus 7
	Target-Android 4.2.2 – API Level 17
	CPU/ ABI- Intel Atom(x86)
	RAM-512 MiB
	SD Card-200 MiB
Experimental features	Static and Dynamic features have been extracted from APK file
	Static features- Permission, API calls, version, services, library, broadcast receivers
	Dynamic features- Battery Charging, Battery Temperature, Network traffic, Memory, CPU, SMS
Data source location	Indira Gandhi Delhi Technical University for Women, Delhi, India (28.6653° N, 77.2324° E)
Data accessibility	Data are in repository with limited access
Related research article	Shivi Garg, Niyati Baliyan, A.K. Mohapatra, A Novel Parallel Classifier Scheme for Vulnerability Detection in Android. Computers & Electrical Engineering. 2018 Dec. “Ready for decision”.

**Value of the data**

• Android APKs can prove to be an effective tool to detect malware in application. Different supervised machine learning algorithms can be used to detect malicious and benign application. We presented an analysis framework that can detect malwares in Android apps using supervised machine learning classifiers.
• In addition to supervised algorithms, several unsupervised and deep learning methods can be applied on this APK data set to classify the Android malware in different families having different functionalities and properties.
• This APK data set can be used by Android App developers and software engineers to devise new data analysis methods and various visualization tools in order to evaluate maliciousness in the android application.
• The dataset presented in this data article can be used to reproduce the results in the research article entitled “A Novel Parallel Classifier Scheme for Vulnerability Detection in Android” [1].

## 1 Data

Malicious and benign APK (~85,000) data from four different datasets- Google Play store [2], Wandoujia [3], AMD [4] and Androzoo [5] are presented in this data article. There are 3 data files included in this data article. AMD contains ~25,000 samples from 2010 to 2016. These are categorized in 135 varieties among 71 malware families. This entire data set is ported into an excel file “Android AMD Malware family data.csv”. Static features of Android APKs – Permissions, Versions, Services, Broadcast Receivers and Libraries are stored in the “staticFeatures.csv”. Binary file named “finalBinary.csv” is created for the static and dynamic features, where 1 represents presence of a particular feature and 0 represents the absence of that particular feature. This file will be considered as a training dataset fed to the machine learning algorithms for performance matrix generation.

**Fig. 1:**
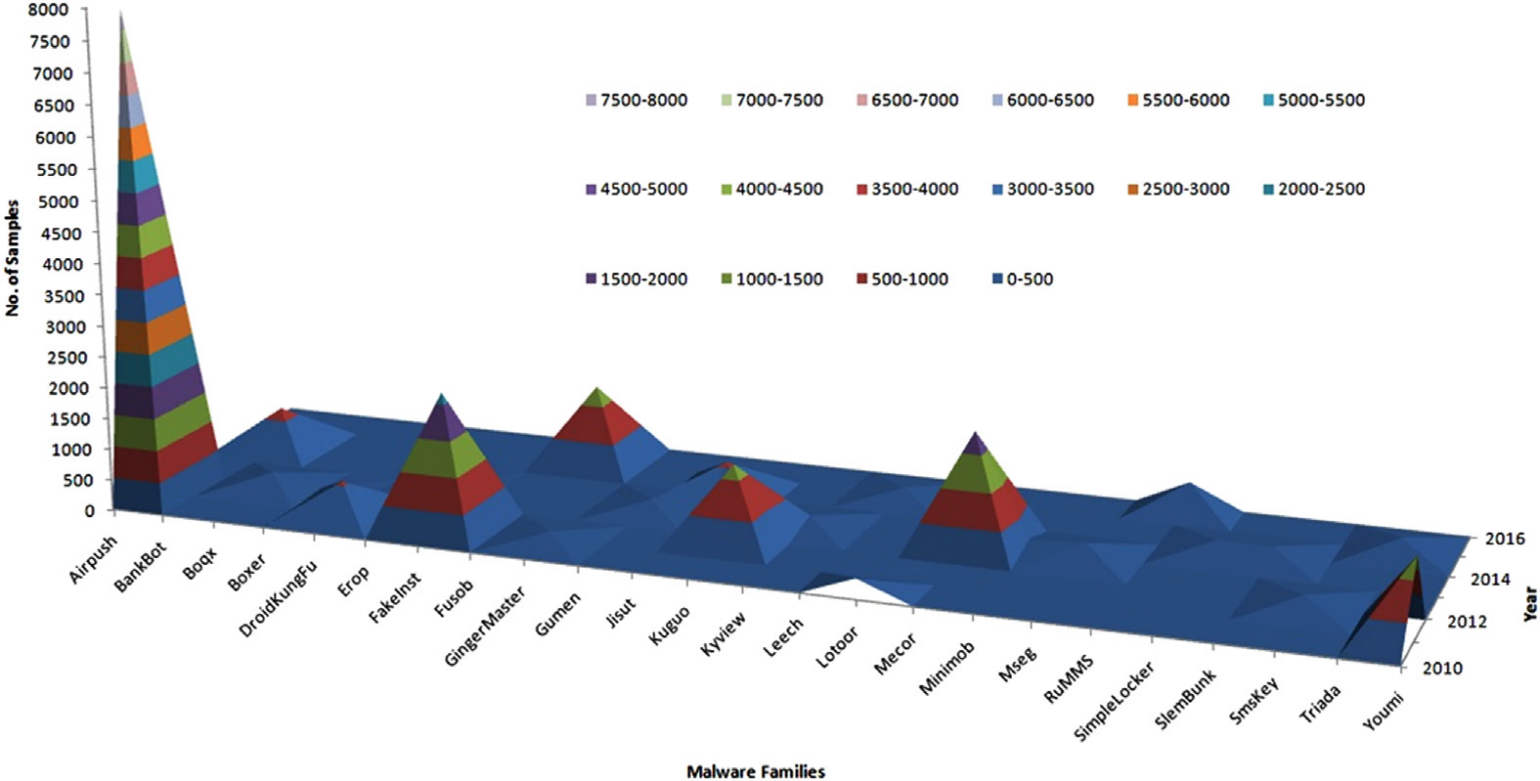
Surface distribution of malware families and creation date.

**Fig. 2:**
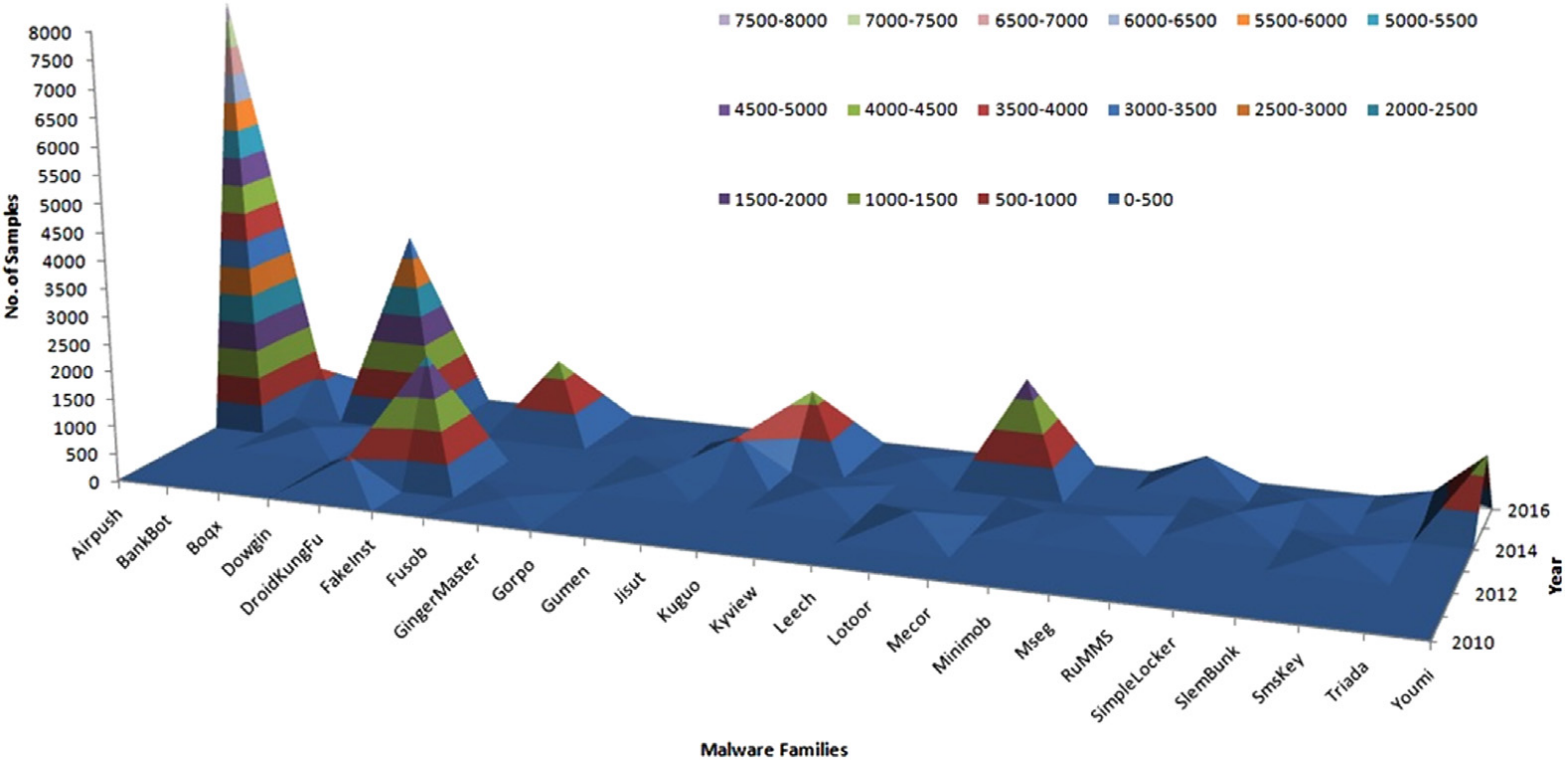
Surface distribution of malware families and detection date.
iv) To access AMD data set:
  SSH Key is shared by AMD for downloading APKs on the local machine in accordance to the access policy [8].
v) Each APK file has six attributes- SHA256, SHA1, MD5, APK Size, Market and Certificate.
vi) APKs are decompiled to Manifest, Gradle, and Smali files using a reverse engineering tool called APKTool [9]. To decompile an APK, use the following command:
  *$apktool d APK_name.apk*
vii) Using Python script, static and dynamic features are extracted using Bag of Words(BoW) feature extraction algorithm
viii) For static features:
  a) Androids manifests provide permissions, broadcast receivers, and services.
  b) Gradle provides version and library used.
  c) Smali provides API calls.
ix) Bag of Words (BoW) feature extraction algorithm: Figs. 3 and 4
  a) Dalvik executable file (.dex) is extracted from an APK file.
  b) .dex files are then transformed to java archive.
  c) .class files are then extracted from the java archive to create .java files.
  d) All java source code files are then merged to form a single source file of the same APK.
  e) Java source code file containing function calls and arguments, import statements and instructions, is represented as bag-of-words.
  f) Source code is then tokenized into unigrams.

i) To download an APK from Google play store and Wandoujia app market, we used an online APK downloader called APKPure.com [6].
ii) To access Androzoo [7] data set:
  To download a benign APK from Androzoo using web Browser (Firefox, Chrome, etc.), use the following command in the web browser https://androzoo.uni.lu/api/download?apikey=${APIKEY}&SHA256=${SHA256}.
iii) AMD contains dataset ~25,000 malicious APK samples from 2010 to 2016. These are categorized in 135 varieties among 71 malware families. Figs. 1 and 2 shows the distribution of malware families with creation date and detection date respectively.

See Figs. 5 and 6.

**Fig. 3:**
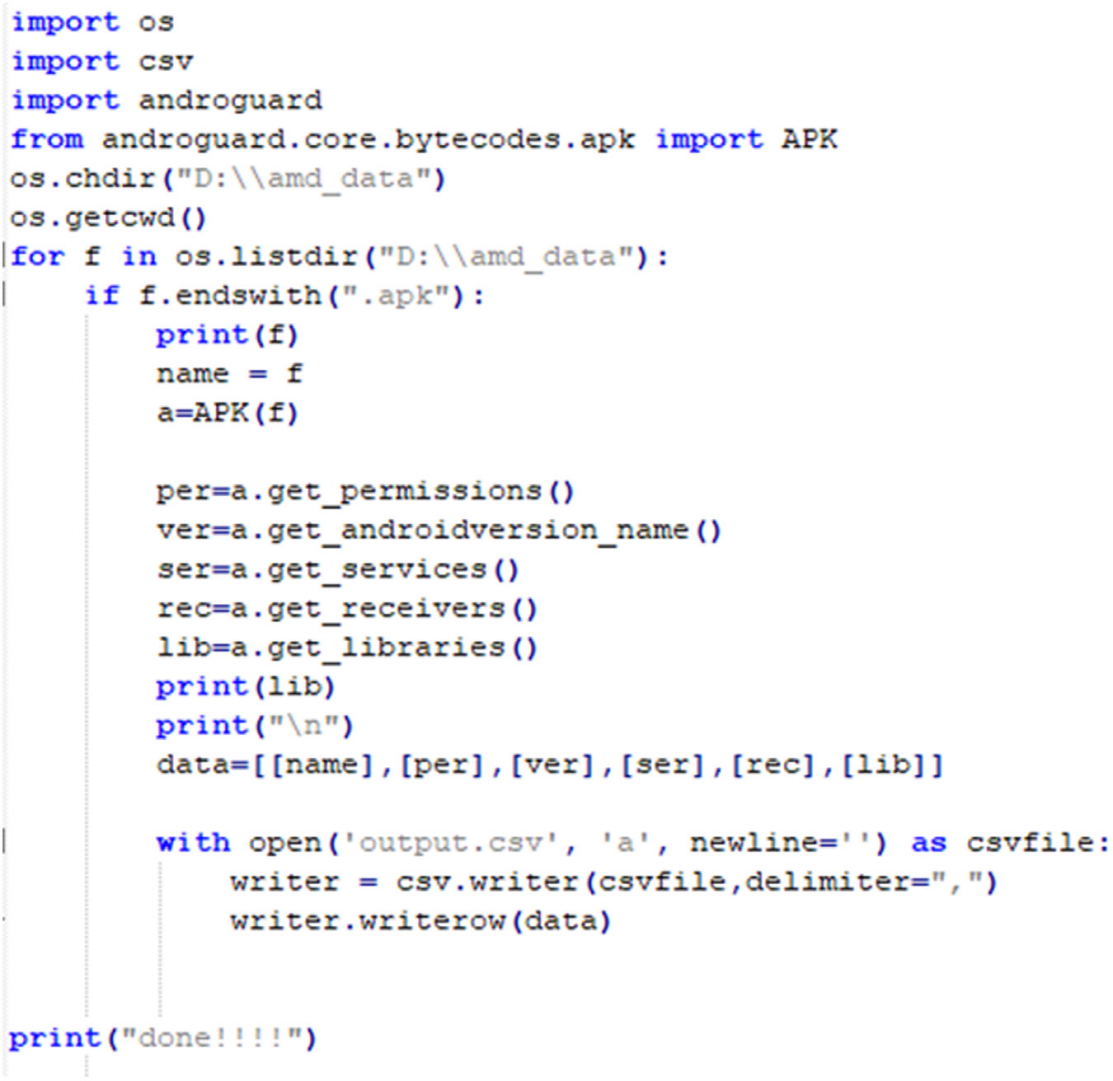
Code snippet to capture attributes of Android APK.

**Fig. 4:**
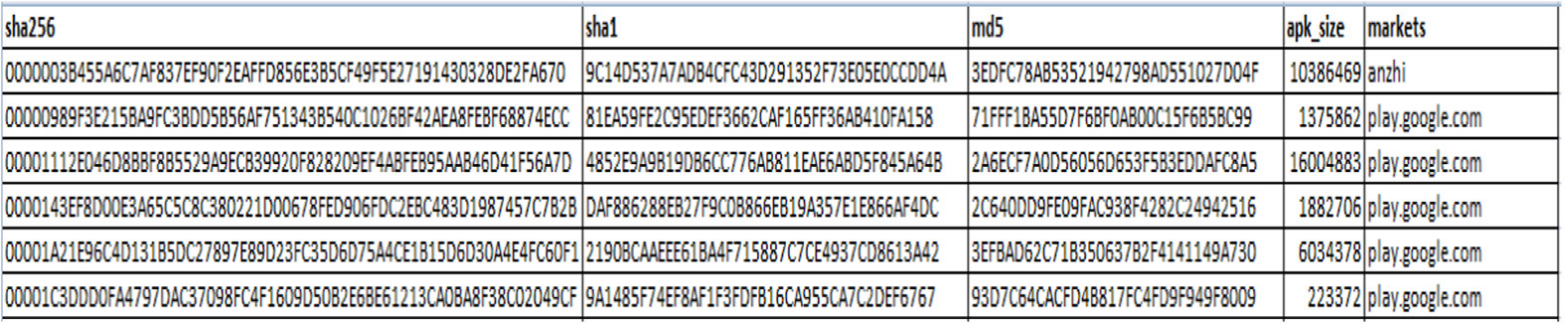
Partial snapshot of the Android APK attributes.

**Fig. 5:**
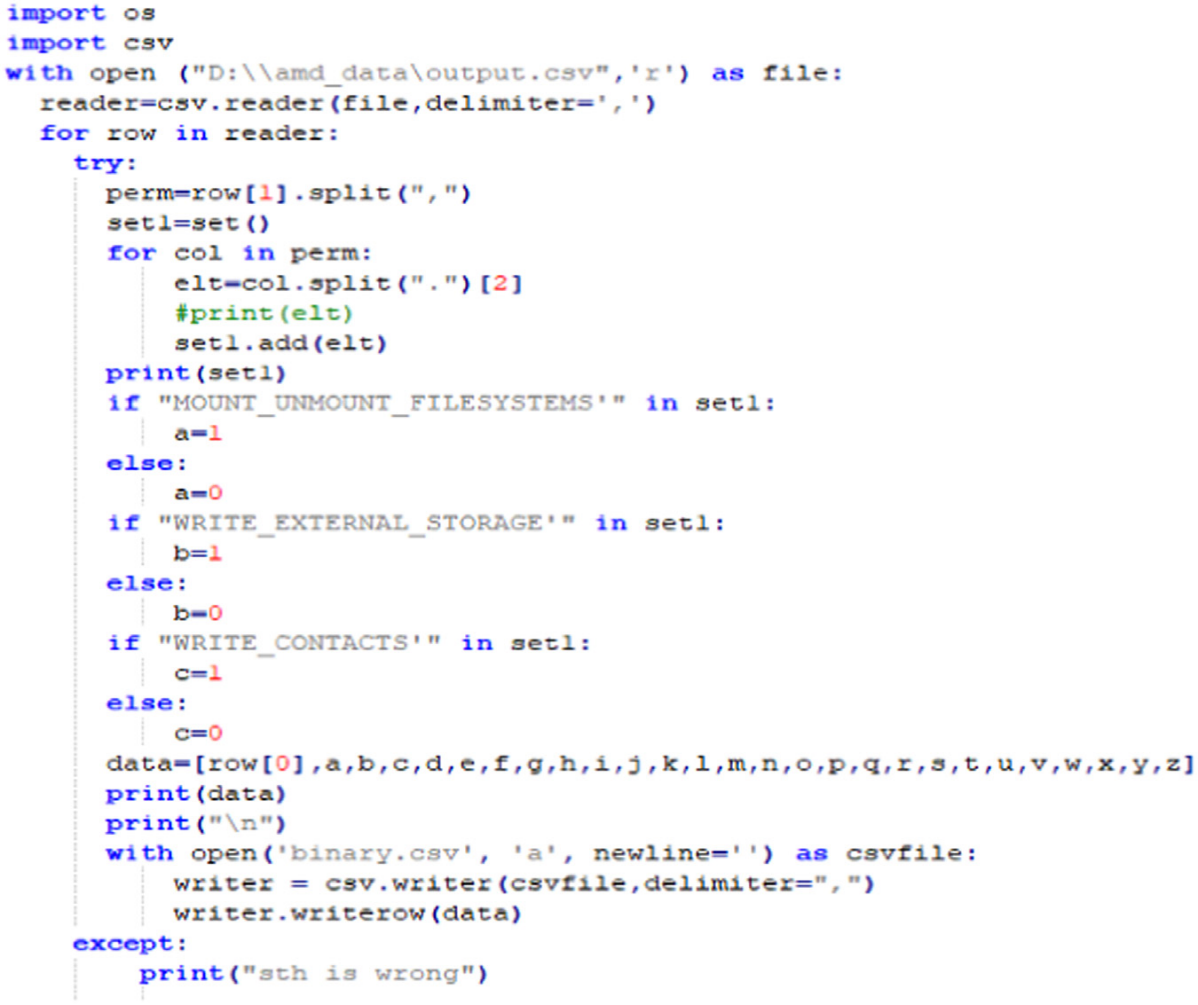
Code snippet of feature extraction.

**Fig. 6:**
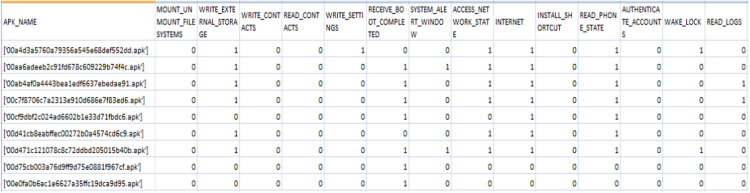
Partial snapshot of the Android features.

## 2 Experimental design, materials, and methods

Different tools have been used to carry out this process. APKPure tool was used to download APKs from Google play store and Wandoujia app market. APKtool was used for reverse engineering APK files. These APK files are decompiled into Manifest, Gradle, and Smali files to extract the static features. For dynamic feature extraction, in-built emulator in Android studio [10] was connected with the Android Debug Bridge (ADB) shell (a command line tool). To capture the random events like-system level events, gestures, touch, keyboard strokes, etc., monkey tool [11] is made to run on ADB shell of the emulator. Feature vector is then created using the static and dynamic feature which is then fed to machine learning algorithms to detect the malware classification accuracy. Specific system configuration is needed for this experiment to be carried. Table 1 summarizes the configuration details of the host and the guest machines.

**Table 1:** Configuration details of Host and Guest machine.

Host machine	
Model	Dell latitude e5250
Processor	Intel(R) Core™ i5–5300U CPU @ 2.30 GHz 2.29 GHz
RAM	16.0 GB
System type	64-bit operating system
Operating system	Windows 10
Guest machine	
Operating system image	Ubuntu 14.04 LTS
Memory	226.0 GB
System type	32-bit operating system
Android emulator configuration	
Platform	Android studio 1.5.1
Device	Nexus 7
Target	Android 4.2.2–API Level 17
CPU/ABI	Intel Atom(×86)
RAM	512 MiB
SD card	200 MiB

Here, supervised machine learning classifiers like Multilayer perceptron (MLP), Support Vector Machine (SVM), Pruning Rule-based Classification Tree (PART) and Ripple down Rule Learner (RIDOR) are implemented on the feature vector to detect the malware in Android APK. Further ensemble techniques such as- Average probabilities, Product of Probabilities, Maximum Probabilities and Majority voting are used to improve the detection rate as compared to that of individual classifiers.
